# 

**Published:** 1841

**Authors:** 


### CONTENTS OF NOS. IX & X.

ART. I. W. W. H. Thackston's Letter on the Turnkey, 185.

II. Important announcement, 189.

III. Information concerning the American Society of Dental Surgeons. By Solyman Brown, M. D. 190.

IV. Notice. 196.

V. Our Next Volume. 197.

VI. Introductory Lecture, delivered before the Class of the Baltimore College of Dental Surgeons, at the opening of its first Session. By C. A. Harris, M. D., D. D. S., Professor of Practical Dentistry. 198.

VII. Asphyxia, or the appearances of the Teeth of those who have died from Strangulation. 212.

VIII. Neuralgia Facialis. By. S. Grandin. 214.

IX. Letter from Dr. Harris to the New-York Editor. 217.

X. Doctor Crane's Letter. 219.

XI. Letter to the Baltimore Editor, from J. C. McCabe, of Richmond, Va. 225.

XII. Old German Remedy for the Tooth-ache. 226.

XIV. Cure of Tooth-ache—destruction of Living Nerve, and removal of the tenderness of the bony cavity of a decayed tooth, without pain in most cases, and with slight pain in any. By Solyman Brown, M. D. 227.

XV. On the development and Structure of the Teeth. 228.

XVI. Miscellaneous Notices. 239.

XVII. On the use of Asbestos, in stopping a certain class of carious teeth.— By Solyman Brown, M. D. 241.

XVIII. American Society of Dental Surgeons. 242.

XIX. Review. 243.

XX. Our Subscription. 244.

XXI. Correction. 246.

XXI. Valuable Reprint. 247.

XXII. Notice of Stockton's Establishment. 248.

